# R-spodin2 enhances canonical Wnt signaling to maintain the stemness of glioblastoma cells

**DOI:** 10.1186/s12935-018-0655-3

**Published:** 2018-10-11

**Authors:** Si Liu, Kin Pong U, Jieting Zhang, Lai Ling Tsang, Jiawei Huang, Shui Ping Tu, Xiaohua Jiang

**Affiliations:** 1 Key Laboratory for Regenerative Medicine of the Ministry of Education of China, School of Biomedical Sciences, Faculty of Medicine, The Chinese University of Hong Kong, Hong Kong, SAR People’s Republic of China; 2 School of Biomedical Sciences Core Laboratory, Shenzhen Research Institute, The Chinese University of Hong Kong, Shenzhen, People’s Republic of China; 3 Department of Oncology, Renji Hospital, School of Medicine, Shanghai Jiaotong University, Shanghai, China

**Keywords:** R-spondin2, Glioblastoma, Stemness, Wnt, Cancer stem cells

## Abstract

**Background:**

As newly identified Wnt enhancer, R-spondin gene family members have been linked to various cancers; however, their role in isocitrate dehydrogenase-wildtype subtype of human glioblastoma (GBM) cells remains unknown.

**Methods:**

Human U87 and U251 cell lines were used to perform the experiments. GBM stem-like cells were enriched in stem cell growth media and induced to differentiate using retinoid acid or growth factor deprivation. Wnt^high^ and Wnt^low^ subpopulations were isolated and evaluated by MTS, sphere formation, transwell migration and xenograft formation assays.

**Results:**

R-spondin 2 but not R-spondin 3 potentiates Wnt/β-catenin signaling in GBM cell lines. While R-spondin 2 does not affect cell growth, it induces the expression of pluripotent stem cell markers in combination with Wnt3A. GBM stem-like cells are endowed with intrinsic high activity of β-catenin signaling, which can be further intensified by R-spondin 2. In addition, R-spondin2 promotes stem cell self-renewal and suppresses retinoid acid- or growth factor deprivation-induced differentiation, indicating R-spondin 2 maintains stem cell traits in GBM. On the other hand, we identify subpopulations of GBM cells that show distinctive responsiveness to Wnt/β-catenin signaling. Interestingly, Wnt^high^ and Wnt^low^ cells display distinctive biologic properties. Moreover, Wnt^high^ cell-inoculated xenografts exhibit enhanced tumorigenicity and increased expression levels of R-spondin 2 compared to Wnt^low^ cell-inoculated xenografts.

**Conclusion:**

Our study reveals a novel regulatory mechanisms underlying the over-activation of β-catenin-mediated signaling in the pathogenesis of GBM.

**Electronic supplementary material:**

The online version of this article (10.1186/s12935-018-0655-3) contains supplementary material, which is available to authorized users.

## Background

Glioblastoma multiforme (GBM), one of the most devastating and lethal forms of human cancer, has a median survival rate of only about 15 months [1]. In recent years, so-called cancer stem cells (CSCs) have been isolated from human tumors [2]. These cells normally constitute a minority population and are proposed to be the cells from which tumors are derived and maintained. The corollary of the existence of CSCs is that tumors are not homogeneous but rather, are comprised of a heterogeneous assortment of abnormally differentiated cells derived from an abnormal clonal stem cell pool [3]. The glioblastoma stem cells (GSCs) have been identified in GBM and are likely responsible for the failure of treatment and high recurrence rates [4]. GSCs are capable of self-renewal and differentiation, and de novo tumor formation when implanted in xenograft models [5, 6]. Furthermore, GSCs possess unique surface markers, such as CD133, SSEA-1, Nestin and OLIG2 [5, 7–9], some of which modulate characteristic signaling pathways and play key roles in GBM vascular formation [10, 11].

It has been well established that Wnt/β-catenin pathway contributes to cancer development since the discovery that Wnt1 was capable of promoting breast cancer 30 years ago [12]. This recognition has been largely strengthened by the findings that intracellular components of the Wnt/β-catenin signaling cascade such as *APC*, *Axin*, and *β*-*Catenin (CTNNB1)*, are mutated in a large variety of human cancers [13]. Besides the numerous mutations found in the pathway components, the contribution of various family members of Wnt ligand has also been recognized in human cancers [14]. Notably, therapeutic agents, which target the binding process of Wnt ligands, have been shown to suppress tumor growth in various xenograft models [15–19]. For instance, OMP-18R5 (vantictumab), an antibody that blocks Wnt binding to 5 out of the 10 human Frizzled receptors, inhibits the growth of a wide range of human cancers [17], indicating that extracellular Wnt signals play an essential role in cancer development. Compared to other cancers, the role of Wnt/β-catenin signaling in GBM is less clear. While aberrant activation of Wnt/β-catenin signaling has been involved in the pathogenesis of GBM [20, 21] and β-catenin is a predictive marker of short survival in GBM patients [22], most GBM do not harbor driver mutations in the canonical Wnt signaling pathway. Thus, it is conceivable that other regulatory mechanisms that trigger the over-activation of β-catenin-mediated signaling are involved in the pathogenesis of GBM [23, 24].

The R-spondin (Rspos) thrombospondin type 1 repeat (TSR1)-containing protein superfamily contains four secreted proteins Rspo1–4 [25], which are emerged as important stroma-derived growth factors driving the renewal of epithelial stem cells in many adult vertebrate tissues [26]. Lgr4–6 are selectively expressed in various tissue stem cells [27] and are the primary high-affinity receptors for Rspos [28]. Mechanistically, Rspos markedly amplify target cell sensitivity to Wnt ligands by neutralizing two transmembrane E3 ubiquitin ligases, Znrf3 and Rnf43, which reduce cell-surface levels of Wnt receptors from internalization and degradation [29, 30]. Of note, Rspos have been emerging as important regulators of cancer development in recent years. *Rspo2* and *Rspo3* were firstly identified as sites of integration for MMTV-induced mammary tumors in mice, which suggest a role of *Rspos* as breast cancer oncogenes [31]. Subsequently, genomic rearrangements that result in elevated RSPO expression have been identified in human colon cancers and demonstrated to activate Wnt signaling and tumorigenesis [32, 33]. More interestingly, it was shown recently that Rspos promoted CSC traits [34], whereas therapeutic targeting on Rspos induced stem cell differentiation [35, 36]. However, it should be noted that contradictory findings have also been reported by various studies. In colon cancer, R-spondin 1 and R-spondin 2 have been demonstrated to suppress CRC tumorigenesis and progression via Wnt-dependent or-independent mechanisms [37, 38]. Taken together, while Rspos have been identified as Wnt enhancers and implicated in cancer development, the exact role of Rspos in cancer development is still controversial.

Given that aberrant activation of Wnt/β-catenin pathway has been implicated in GBM development, we hypothesized that Rspos might modify canonical Wnt signaling in GBM cells, and be indicative of cancer stemness trait regulation. We undertook the present study in two different human isocitrate dehydrogenase (IDH)-wildtype subtype glioblastoma cell lines, U251 and U87, to determine the role of Rspo family members in GBM.

## Materials and methods

### GBM cell culture and reagents

Both of U87 and U251 cell lines are IDH-wildtype subtype of glioblastoma (GBM-IDH-wt) according to the recent change in classification of gliomas. U87 and U251 were purchased from ATCC (Manassas, VA, USA). The authentication of the U251 and U87 cell lines have been tested by short tandem repeat (str) profiling by Department of Pathology, the Chinese University of Hong Kong in 2016 and 2017. Cells were cultured in DMEM medium (Gibco Invitrogen, Grand Island, New York, USA) supplemented with 10% fetal bovine serum (Gibco) and 1% penicillin–streptomycin, at 37 °C and 5% CO_2_. Cells were maintained in T75 flasks or T25 flasks, and passaged every 2–3 days when reached 70–80% confluence. Recombinant Murine Wnt3A, human R-spondin 2 and R-spondin 3 were purchased from PeproTech (Rocky Hill, NJ).

### GSC culture

The following GSC culture medium was applied to enrich GSCs: DMEM/F12 medium (Thermo Fisher Scientific, Grand Island, NY) supplemented with 1 × B-27™ Supplement, serum free (Gibco, 17504044), 20 ng/ml Animal-Free Recombinant Human EGF (PeproTech, AF-100-15), 20 ng/ml Recombinant Human FGF-basic (154 a.a.) (PeproTech, 100-18B), and 1× penicillin–streptomycin. Cells were seeded in Costar^®^ 24 Well Clear Flat Bottom Ultra Low Attachment plates (Corning, NY) at 1000 cells/ml. Spheres were carefully aspired into 15 ml tube, and spin down at 1000 rpm for 3 min. Subsequently, 1 ml warm trypsin was used to digest spheres at room temperature for 3 min. The reaction was stopped by adding 10 ml GSC culture media, then single cells were spin down and re-plated.

### Lentiviral transduction

7TGP vector was purchased from Addgene (#24305). This is a lentiviral GFP-coupled Wnt reporter construct constrains a GFP gene under the control of 7 TCF responsive element, which yields expression of GFP only in cells with activated Wnt/β-catenin signaling (Additional file 1: Fig S1C). In brief, 5 × 10^6^ 293T cells were seeded in 10-cm dishes 1 day before transfection. For each dish, 8 μg of 7TGP lentiviral vector were mixed with 3 μg of the VSV-G envelope plasmid (pMD2.VSVG) and 6 μg of the packaging plasmid (pCMVDR8.74). The solution was topped up to 250 μl with water and mixed with 250 μl 0.5 M CaCl_2_. The precipitate was formed by adding 500 μl of 2 × HEPES-buffered saline (280 mM NaCl, 10 mM KCl, 1.5 mM Na_2_HPO_4_, 12 mM dextrose, 50 mM HEPES, pH7.2) drop-wise while vortexing and added directly to the cells. The medium was replaced after 16 h and conditioned twice for 24 h.

### Analysis of Wnt/β-catenin activity and isolation of Wnt^high^ and Wnt^low^ cell population

U251 cells were transduced with 7TGP and selected by 2 µg/ml puromycin for 1 week. The cells were then cultured in serum-free DMEM medium for 24 h. Then, the cells were treated with serum-free DMEM medium supplemented with different WNT ligands for another 24 h. Cells were washed by PBS and then trypsinized into single cells, analyzed by Flow Cytometer (BD LSRFortessa Cell Analyzer). To enrich Wnt^high^ or Wnt^low^ cells in response to WNT3A, cells were treated in serum-free DMEM medium supplemented with 20 ng/ml WNT3A for 24 h. After 24 h, cells were washed by PBS and sorted by Flow Cytometer (BD FACSAria II Cell sorter) in FITC channel. Wnt^high^ cells were sorted from the highest 5% GFP^+^ cells and Wnt^low^ cells were sorted from the lowest 5% GFP^+^ cells. After sorting, cells were changed back to normal GBM medium, and allowed to grow for another two passages. Afterward, the sorting was repeated for 4–5 times.

### MTT assay

3000–6000 cells were seeded in 200 µl medium in a well of a 96-well plate. Cells were cultured to 60% confluence in normal medium, then medium was changed to serum-free DMEM medium 24 h before experiment began. After that, cells were cultured in serum-free DMEM medium containing different Wnt ligands (all at 20 ng/ml). MTT assay were performed according to the protocol recommended by the Vendor.

### Holoclone assay

Cells were trypsinized into single cells and seeded at 100–500 cells per well in 6-well plate. Cells were cultured for 14 days, and medium was changed every 3 days. At the end of the experiment, cells were fixed by methanol at room temperature and stained with 0.5% Crystal Violet for 5 min at room temperature. Colonies were classified into holoclones, meraclones and paraclones according to their morphology.

### Soft agar assay

The lower layer of agar was mixed by equal volume of 2× DMEM medium containing 1.2% agar solution. This mixture was added into each well of a 6-well plate immediately. The upper layer of agar was carefully mixed by adding equal volume of 2× DMEM medium and 0.6% agar solution, as well as 3000–8000 cells. 1.5 ml normal glioma medium was then added in each well, and medium was changed every 3 days for 10–14 days. At the end of experiment, cells were fixed by methanol at room temperature and stained with 0.5% Crystal Violet for 5 min. The plate was scanned, from which colony numbers were counted.

### Sphere formation assay

2000–3000 single cells were cultured in GSC medium in one well of an ultra-low attachment 6-well plate. Medium was changed every 3 days by centrifuging spheres at 1000 rpm for 2 min. Spheres were counted after 10–14 day culture. To determine the self-renewal ability, spheres were trypsinized and replated at the concentration of 1000 cells/ml for another 10 days. After that, spheres were fixed by 70% ethanol, both sphere numbers and diameters were calculated.

### Transwell cell migration assay

Cells were allowed to grow to 70–80% confluence, then treated with serum-free DMEM medium for 24 h. Then, cells were trypsinized into single cells and counted. 2 × 10^4^ cells were seeded in the upper chamber whereas 0.5 ml DMEM medium with 10% FBS, or Wnt ligands was added in the lower chamber. Cells were allowed to migrate into the lower well for 24 h. After 24 h, upper wells were fixed in methanol for 5–10 min at room temperature, and then stained with 0.5% Crystal Violet for 5 min. In each group, triplicates were used.

### RA induced neural differentiation

3000 GSCs were allowed to form neurospheres for 10 days (GSC medium was changed every 3 days), then the spheres were seeded in DMEM serum-free medium containing 10 µm RA in the presence or absence of Wnt ligands for 24–48 h. In addition, one group without RA was used as control representing undifferentiated state.

### FACS analysis of CD133 expression

In brief, cells were digested with either trypsin (for adhesive cells) or Cell Dissociation Buffer (enzyme-free, Thermo Fisher, for suspended cells). Then, 1:20 CD133-APC conjugated antibody was dissolved in binding buffer. Up to 1 × 10^6^ cells were suspended with antibody in 200 µl binding buffer at 4 °C for 15 min in dark. After antibody incubation, cells were washed twice by PBS at 1000 rpm for 3 min. Then the cells were analyzed by Flow Cytometer in APC channel. Mouse IgG-APC was used as negative control.

### Quantitative real-time PCR

TRIzol Reagent (Thermo Fisher Scientific) was used to extract total RNA. High-Capacity cDNA Reverse Transcription Kit (Invitrogen) was used to synthesize cDNA from 2 μg RNAs per reaction (20 μl), according to manufacturer’s instruction. For real-time assay, miScript SYBR Green PCR Kit (Qiagen, Germantown, MD) was strictly applied according to manufacturer’s instruction. ABI QuantStudio 7 (QS7) Flex Real Time PCR System (384-well) was used for amplification. Real-time data was analyzed by ABI QuantStudio 7 (QS7) Flex Real Time PCR System Station. Primer used were summarized in Additional file 2: Table S1.

### Western blot

Protein samples were extracted by RIPA buffer, and 60 μg was separated on a 10% SDS–PAGE gel which subsequently transferred onto a PVDF membrane (Sigma-Aldrich). After blocking with 4% milk at room temperature for 1 h, the membrane was incubated with primary antibody overnight at 4 °C on a horizontal rotor. Membrane was washed three times with TBST and incubated with secondary antibodies at room temperature for 1 h. Later, the blot was subjected to chemiluminescent detection with ECL Detection Reagent (Amersham GE Care), and was scanned for analysis. Antibodies used were summarized in Additional file 3: Table S2.

### Immunohistochemistry staining

Tumor tissues were fixed in 4% paraformaldehyde PBS solution at 4 °C for 24 h and then embedded in paraffin. Tissues were cut into 6 μm sections and de-paraffined three times in xylene and rehydrated in gradient alcohols. Endogenous peroxidase activity was quenched with 3% H_2_O_2_ in PBS for 30 min at room temperature, and sections were washed in PBS 5 min for three times. The sections were heated by microwave at 98 °C for 20 min in 10 mM citrate buffer (pH 6.0) for antigen retrieval (PT Module, Thermo). Sections were blocked with 5% horse serum for 30 min at room temperature and incubated with primary antibodies at 4 °C overnight. Later, radish peroxidase-conjugated secondary antibodies (rabbit or mouse, Santa Cruz) were incubated at room temperature for 1 h. Sections were developed with diaminobenzidine and counterstained with hematoxylin using standard protocol.

### Xenograft formation

Nude mice were provided by the Laboratory Animal Service Center of the Chinese University of Hong Kong. They were maintained in an air-conditioned room with controlled temperature of 24 ± 2 °C and humidity of 55 ± 15%, in a 12 h light/darkness cycle regulation and were fed laboratory chow and water ad libitum. All animal experiments were conducted in accordance with the University Laboratory Animals Service Center’s guidelines on animal experimentation with approval from the Animal Ethnics Committee of the University. Female nude mice between 4 and 6 week-old were used in the experiments. 100 µl PBS containing different cell numbers (from 1 × 10^4^ to 1 × 10^6^) was injected at the flanks of the mice. After inoculation, the condition of nude mice was checked on a daily basis, and tumor volume was recorded using formula V = (a × b^2^)/2, where a represents the longest side of tumor and b represents the width of tumor. Mice with tumor volume larger than 1.5 cm^3^ or sign of suffer were sacrificed immediately. At the end of the experiment, mice were sacrificed by CO_2_, and tumors were carefully dissected. Tumor samples were collected for RNA extraction, protein extraction or frozen section.

### Statistical analysis

For in vivo tumor growth curve, one-way ANOVA was used to compare changes in different groups along time. Two-tailed Student’s *t* test was used to compare differences between experimental groups. In each group, data were triplicated or indicated elsewhere, and each bar represents mean ± SD. Stars on each bar represents statistical significance compared to control group, and additional comparisons were indicated with line segments (*p < 0.05, **p < 0.01, ***p < 0.001, ****p < 0.0001, ns for no significant difference).

## Results

### Rspo2 potentiates Wnt/β-catenin signaling in GBM cell lines

**Fig. 1:**
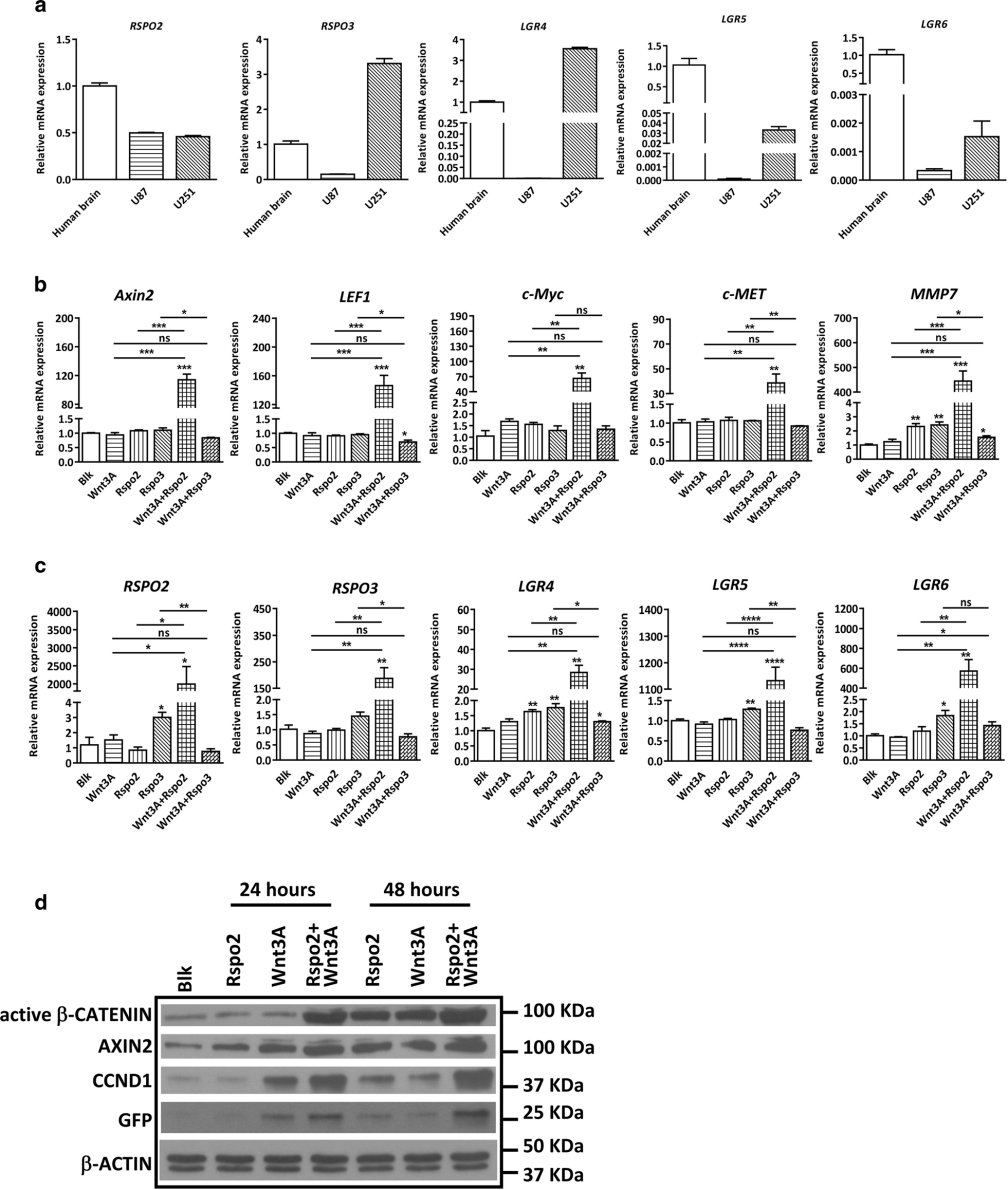
Expression pattern and potentiation effect of *RSPOs* in U251 cells. **a** mRNA expression levels of *RSPO*2/3 and their receptors LGR4–6 were determined in U87 and U251 cells. U251 cells were pre-treated in serum-free medium for 24 h, then cultured in serum-free medium containing different Wnt ligands (20 ng/ml Wnt3A, Rspo2, Rspo3) for another 24 h. Rspo2 shows prominent potentiation effect on Wnt3A-induced canonical β-catenin downstream targets (**b**), and RSPO-LGR genes (**c**). Blk indicates U251 cells cultured in serum-free medium containing 0.1% DMSO. **d** 7TGP-transduced U251 cells were pre-treated in serum-free medium for 24 h, then cultured in serum-free medium containing different Wnt stimuli for another 24–48 h. Representative Western Blot showing the protein expression levels of canonical β-catenin downstream targets in 7TGP-transduced U251 cells with Wnt ligand treatment

We first determined the expression of *RSPO2*, *RSPO3* and their receptors *LGR4*–*6* in GBM cell lines U251 and U87 by real-time PCR. The results showed that while the expression level of *RSPO2* was comparable between U87 and U251, *RSPO3* was more highly expressed in U251. In addition, the expression levels of *LGR4, LGR5* and *LGR6* were higher in U251 than that in U87 (Fig. 1a). Next, U251 or U87 cells were exposed to recombinant Wnt3A, Rspo2, Rspo3 or their combination for 24 h, and examined for the expression levels of β-catenin target genes. Our results showed that while Wnt3A, Rspo2 or Rspo3 alone had no or mild effect on the expression levels of *Axin2*, *LEF1*, *c*-*MET*, *c*-*Myc or MMP7* in U251 cells, the combination of Wnt3A and Rspo2 dramatically up-regulated the expression levels of the β-catenin target genes, indicating Rspo2 potentiates the Wnt3A-mediated activation of β-catenin signaling (Fig. 1b). This induction effect, however, is transient, since neither Rspo2 or Wnt3A alone, nor the combination treatment had any effect at 48 h (data not shown). It should be noted that Rspo3 did not have potentiation effect on Wnt/β-catenin signaling in U251 cells, possibly due to the high intrinsic basal level. In U87 cells, the combination of Rspo2 and Wnt3A significantly enhanced the effect of Wnt3A or Rspo2 alone on *LEF1* and *MMP7* (Additional file 1: Fig S1A), albeit the extent of this effect was much smaller than that in U251 cells.

Additionally, the combination of Wnt3A and Rspo2 dramatically increased the expression of *RSPO2*, *RSPO3* and *LGRs* in U251, with the most profound effects observed in *RSPO2* and *LGR5* (Fig. 1c). Interestingly, while Rspo3 alone mildly increased the expression of *RSPO2* and *LGRs*, the combination of Wnt3A and Rspo3 diminished this effect (Fig. 1c), which is consistent with the effect of Rspo3/Wnt3A on β-catenin targets (Fig. 1b). In U87 cells, while Rspo2 did not have any effect on the expression levels of *RSPO2* and *LGRs*, the combination of Wnt3A and Rspo2 significantly increased the expression levels of *RSPO2* and *LGRs*, albeit at much lower level compared to that in U251 cells (Additional file 1: Fig S1B). To further validate the potentiation role of Rspos in canonical Wnt signaling in GBM cells, we transduced U251 cells with 7TGP lentiviral vector (Additional file 1: Fig S1C) and demonstrated that the combination of Wnt3A and Rspo2 dramatically enhanced the activation of eGFP compared to Wnt3A or Rspo2 alone (Fig. 1d). Consistent with eGFP expression, the expression level of active β-CATENIN was significantly increased in the combination treatment compared to Wnt3A or Rspo2 alone. Moreover, the expression levels of CCND1 and AXIN2 also showed the similar enhancement (Fig. 1d). Altogether, these data clearly demonstrate that Rspo2 enhances Wnt3A-activated β-catenin signaling in GBM cells.

### Combination of Rspo2 and Wnt3A upregulates stem cell genes in GBM cells

**Fig. 2:**
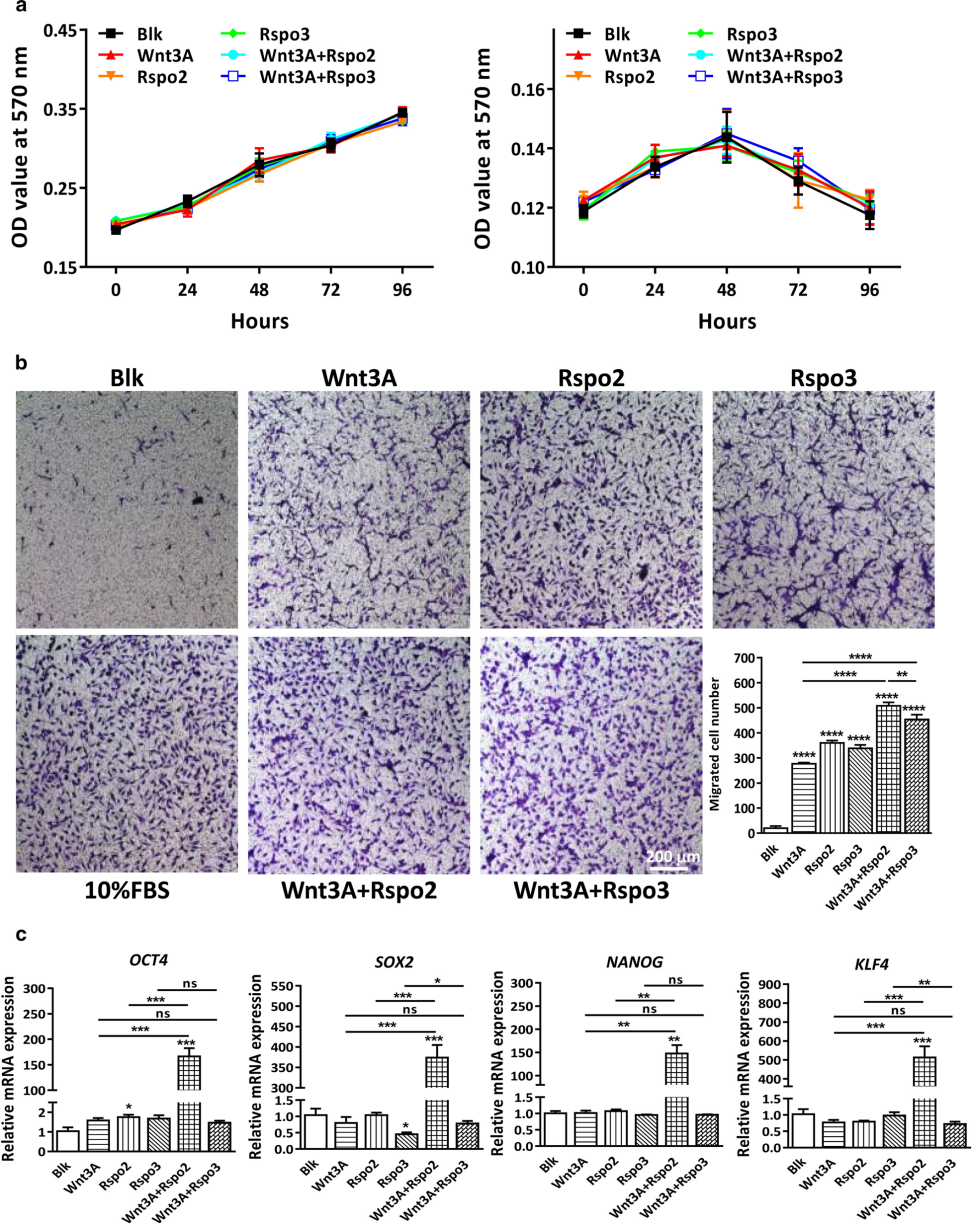
Combination of Rspo2 and Wnt3A does not affect cell growth, but elevates the expression levels of stem cell genes in U251 cells. **a** U87 and U251cells were pre-treated in serum-free medium for 24 h, then cultured in serum-free medium containing different Wnt ligands for another 4 days, and cell growth was determined by MTT assay. Results show neither WNT ligand alone nor in combination has any effect on cell growth. **b** Transwell migration assay was performed in U251 cells. U251 cells were pre-treated in serum-free medium for 24 h, then 2 × 10^4^ cells were cultured in serum-free medium in chambers, while wells containing different Wnt stimuli were used as chemotaxis source for another 24 h. scale bar represents 200 μm. Quantification analysis of data is expressed as the Mean ± SD from triplicates of two independent experiments. **c** The mRNA expression levels of stem cell marker *OCT4*, *SOX2*, *NANOG* and *KLF4* were determined by real time PCR, results show that the combination of Rspo2 and Wnt3A significantly upregulates stem cell markers

Having established that Rspo2 potentiates Wnt/β-catenin signaling in GBM cells, we tried to determine the functional role of Rspo2 in GBM cells. Surprisingly, neither Wnt3A nor Rspos had any effect on cell growth in U251 or U87 cells. The combination of Wnt3A and Rspos did not have any effects on cell growth either (Fig. 2a). We further determined the migratory ability, which is indicative of malignant characteristics of GBM. Interestingly, while Wnt3A or Rspos alone promoted cell migration, the combination treatment significantly aggravated this effect. Consistent with the previous results, the effect of Rspo2 was stronger than Rspo3 (Fig. 2b).

In GBM and other tumors, the expression and regulation of embryonic stem cell markers appear to be critical for the maintenance of tumor-initiating cells and cancer malignancy [39]. Thus, we went further to determine the effect of Rspos on the expression levels of pluripotent markers in GBM cell lines. Our real time PCR showed that while Wnt3A alone did not affect the expression of pluripotent stem cell markers, the combination of Wnt3A and Rspo2 dramatically increased the expression of *OCT4*, *NANOG*, *SOX2* and *KLF4* in U251 cells. In contrast, neither Rspo3 nor Wnt3A/Rspo3 groups had any effects on pluripotent stem cell markers (Fig. 2c). In U87 cells, both Wnt3A and Rspo2 dramatically increased the mRNA expression of *CD133 and SOX2*. However, the effect of Wnt3A or Rspo2 could not be potentiated by the combination therapy. On the other hand, while Wnt3A and Rspo2 alone did not affect *NANOG* expression, the combination of Wnt3A and Rspo2 dramatically increased the expression of *NANOG* (Additional file 1: Fig S2A). Given that GBM are heterogeneous and hierarchically organized with GSCs at their apex, the upregulation of stem cell markers could be due to the enrichment of GSCs or the enhancement of GSC stemness. Our flow cytometry analysis demonstrated that neither Wnt3A or Rspo2 alone nor the combination treatment had any effect on the enrichment of CD133^+^ positive cells in U251 (Additional file 1: Fig S2B), suggesting that Rspo2/Wnt3A enhances stem cell traits in GSCs rather than enlarges the GSC pool within GBM cells.

### Rspo/Lgr and Wnt/β-catenin signaling are over-activated in GSC-like cells

**Fig. 3:**
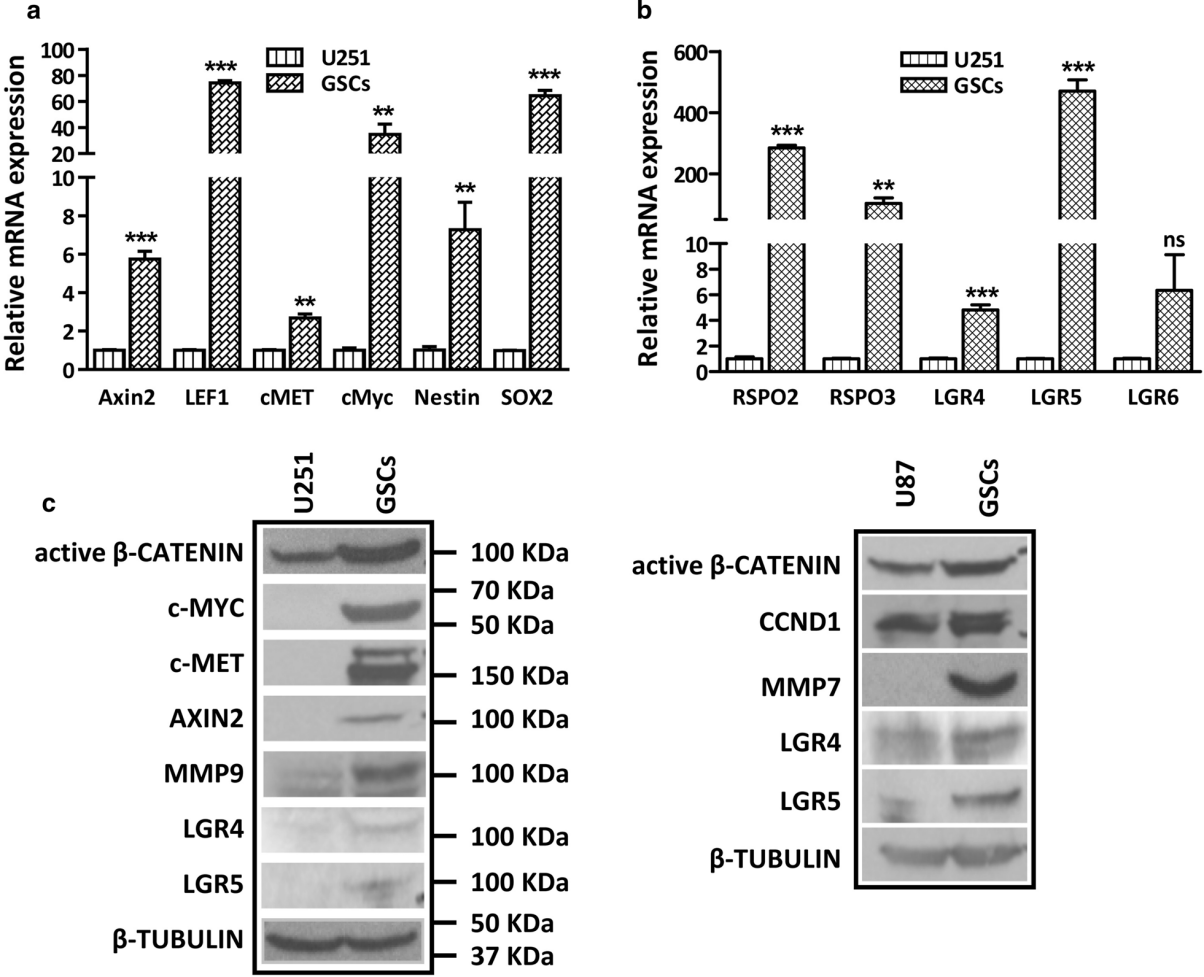
GSC-like cells are endowed with upregulated Wnt-Lgr-Rspo axis. **a** U251 GSCs show dramatic increment of β-catenin targets and neural stem cell markers as determined by real time PCR. **b** Rspo/Lgr axis is upregulated in U251 GSCs as determined by real time PCR. **c** the protein expression levels of β-catenin targets and LGRs were upregulated in U251 and U87 GSCs

Next, we enriched for GSC-like cells from U251 and U87 cell lines using the well-established GSC culture condition [40, 41]. After 2 months of enrichment, we detected the CD133 positivity in U251 and U87 GSC-like cells and found that more than 63% cells were CD133^+^ in U251 whereas 35.3% cells were CD133^+^ in U87 (Additional file 1: Fig S3A). The GSC properties were confirmed by both sphere formation assay and holoclone assay, results of which showed enhanced sphere forming capability and increased number of holoclones in the GSC-like cells (Additional file 1: Fig S3B, C). To determine whether these GSC-like cells are endowed with differential Wnt/β-catenin activity compared to their parental cells, we examined the mRNA expression levels of β-catenin target genes and *RSPOs*/*LGR*s in U251 and U87 GSCs. Our results showed that comparing to U251 parental cells, the mRNA expression levels of β-catenin targets, such as *Axin2*, *LEF1*, *c*-*MET* and *c*-*Myc* were dramatically increased in the GSC-like cells (Fig. 3a). In addition, the expression levels of *Nestin* and *SOX2* were also markedly up-regulated in the GSC population, confirming that GSC-like cells share NSC-associated traits. Besides, the expression levels of *RSPO2*, *RSPO3* and *LGRs* were globally upregulated in U251 GSCs compared to parental cells, indicating that the Rspo/Lgr axis is over-activated in GSC-like cells (Fig. 3b). Consistently, in U87 GSCs, the mRNA expression levels of β-catenin targets and NSC marker, as well as *RSPO2, RSPO3 and LGR4* were markedly increased compared to parental cells (Additional file 1: Fig S3D, E). Next, we determined the protein expression levels of active β-CATENIN, β-catenin target genes and LGRs in U251 and U87 GSCs in relative to their parental cells. In line with the mRNA data, the expression levels of active β-CATENIN and its targets were dramatically increased in the GSC-like cells. In addition, the expression of LGR4 and LGR5 was also significantly increased in the GSC-like cells compared to their parental cells (Fig. 3c).

### Rspo2 and Wnt3A maintains the stemness of GSC-like cells

**Fig. 4:**
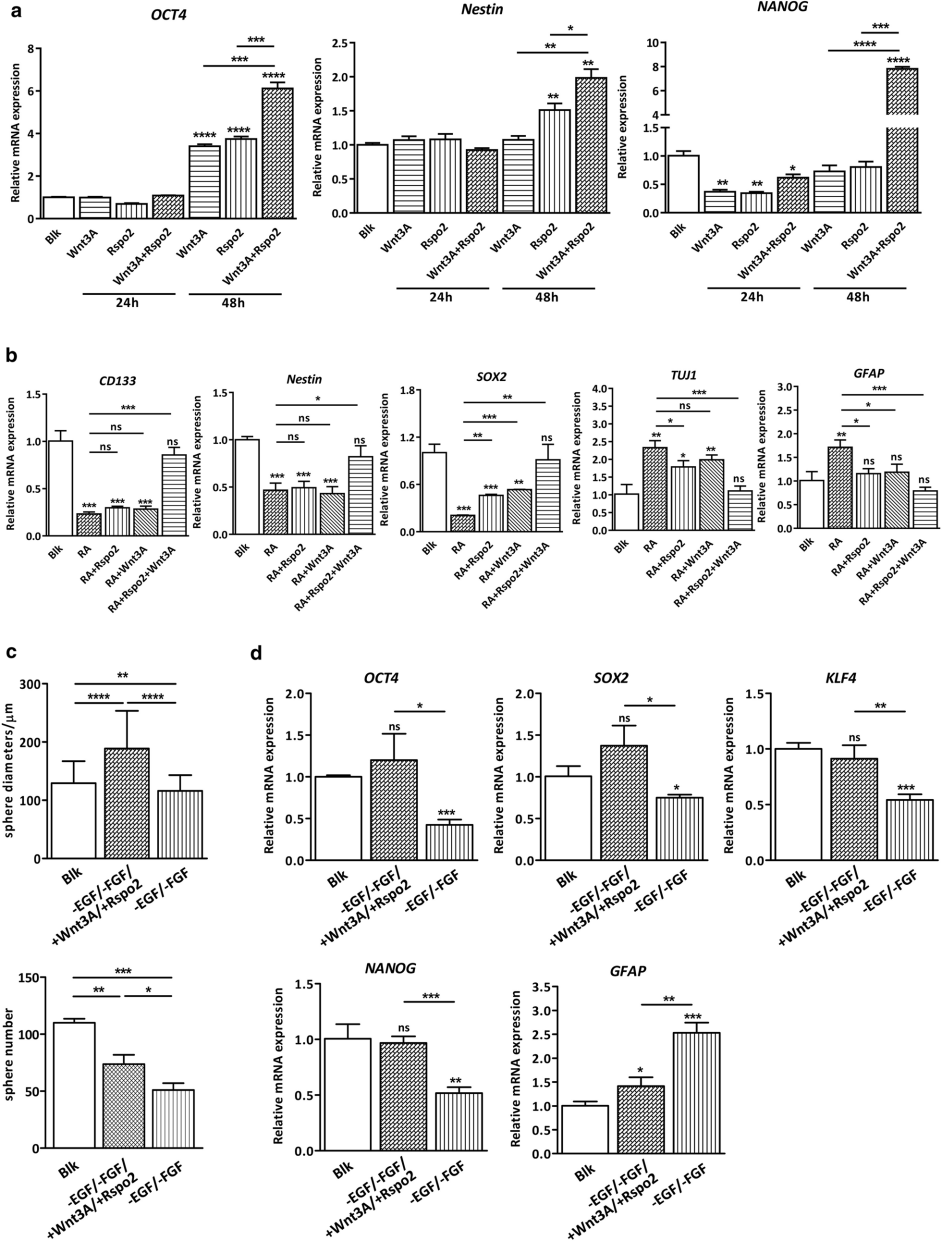
Wnt3A and Rspo2 maintains stemness in GSCs. **a** Rspo2 and Wnt3A upregulates stem cell marker in U251 GSCs as demonstrated by real time PCR. Blk indicates U251 GSCs cultured in GSC media with 0.1% DMSO. **b** All-trans retinoic acid (10 µM RA) was used to induce differentiation in U251 GSCs for 24 h with or without Wnt ligands (20 ng/ml). Real-time PCR was used to determine the effect on differentiation. Results show that Rspo2/Wnt3A treatment rescues RA-induced GSC differentiation. Blk indicates U251 GSCs cultured in DMEM with 0.1% DMSO. **c** GSCs were cultured in GSC media, or GSC media without EGF and FGF, or GSC media without EGF and FGF but with Wnt3A and Rspo2 for 7 days. Results show that lack of EGF/FGF causes reduced sphere formation, which can be compensated by adding Rspo2/Wnt3A. **d** Real-time PCR shows that Rspo2/Wnt3A treatment abolishes the downregulation of stem cell markers and upregulation of differentiation markers caused by growth factor deprivation. Blk in C and D indicates U251 GSCs cultured in GSC media with 0.1% DMSO

Next, we asked what is the role of Rspo2 in GSC-like cells? To answer this question, we evaluated the effect of Wnt3A and/or Rspo2 on stem cell marker induction. As shown in Fig. 4a, while Wnt3A or Rspo2 alone significantly increased the expression of GSC markers such as *OCT4* and *Nestin*, the combination of Wnt3A and Rspo2 aggravated the induction effect. For *NANOG*, neither Wnt3A nor Rspo2 alone increased the expression, however, combined treatment with Wnt3A and Rspo2 dramatically induced the expression. To further demonstrate the role of Wnt3A and Rspo2 in maintaining the stem cell traits of GSCs, we went further to examine the effects of Wnt3A and Rspo2 on preventing neural differentiation of GSCs. In this set of experiments, GSC-like cells were cultured as neurospheres, and then seeded in serum-deprived medium containing 10 µm Retinoid Acid (RA) in the presence or absence of Wnt3A and/or Rspo2. RA treatment induced GSC differentiation robustly as demonstrated by dramatic downregulation of stem cell markers including *CD133*, *Nestin* and *SOX2* and induction of differentiation markers such as *TUJ1* and *GFAP.* Intriguingly, while Wnt3A or Rspo2 alone exhibited mild effect, the combination of Wnt3A and Rspo2 completely rescued the downregulation of stem cell markers and upregulation of differentiation markers (Fig. 4b). The experiments were repeated in U87 GSCs, which revealed the similar results (Additional file 1: Fig S4A). It has been well established that GSCs can be maintained in EGF- and FGF2-enriched culture condition to sustain their stemness. To further consolidate our finding that Wnt3A and Rspo2 is critical for maintaining the stemness of GSCs, we cultured the U251 GSC-like cells without EGF and FGF2 in the absence or presence of Wnt3A and Rspo2. As expected, the deprivation of EGF and FGF2 significantly decreased the diameters and numbers of neurosphere, however, this effect could be significantly reversed by the addition of Wnt3A and Rspo2 (Fig. 4c, Additional file 1: Fig S4B). In addition, grow factor deficiency-induced suppression of stem cell markers could be completely abolished by the co-treatment with Wnt3A and Rspo2 (Fig. 4d). On the other hand, it should be noted that withdrawal of EGF and FGF2 led to a marked suppression of β-catenin signaling (Additional file 1: Fig S4C), which was reversed by the combination of Wnt3A and Rspo2. Altogether, these results clearly demonstrate the essential role of Rspo2 and Wnt/β-catenin signaling in the prevention of GSC differentiation and maintenance of their stemness.

### Wnt/GFP^high^ and Wnt/GFP^low^ cell populations display distinct biologic properties

**Fig. 5:**
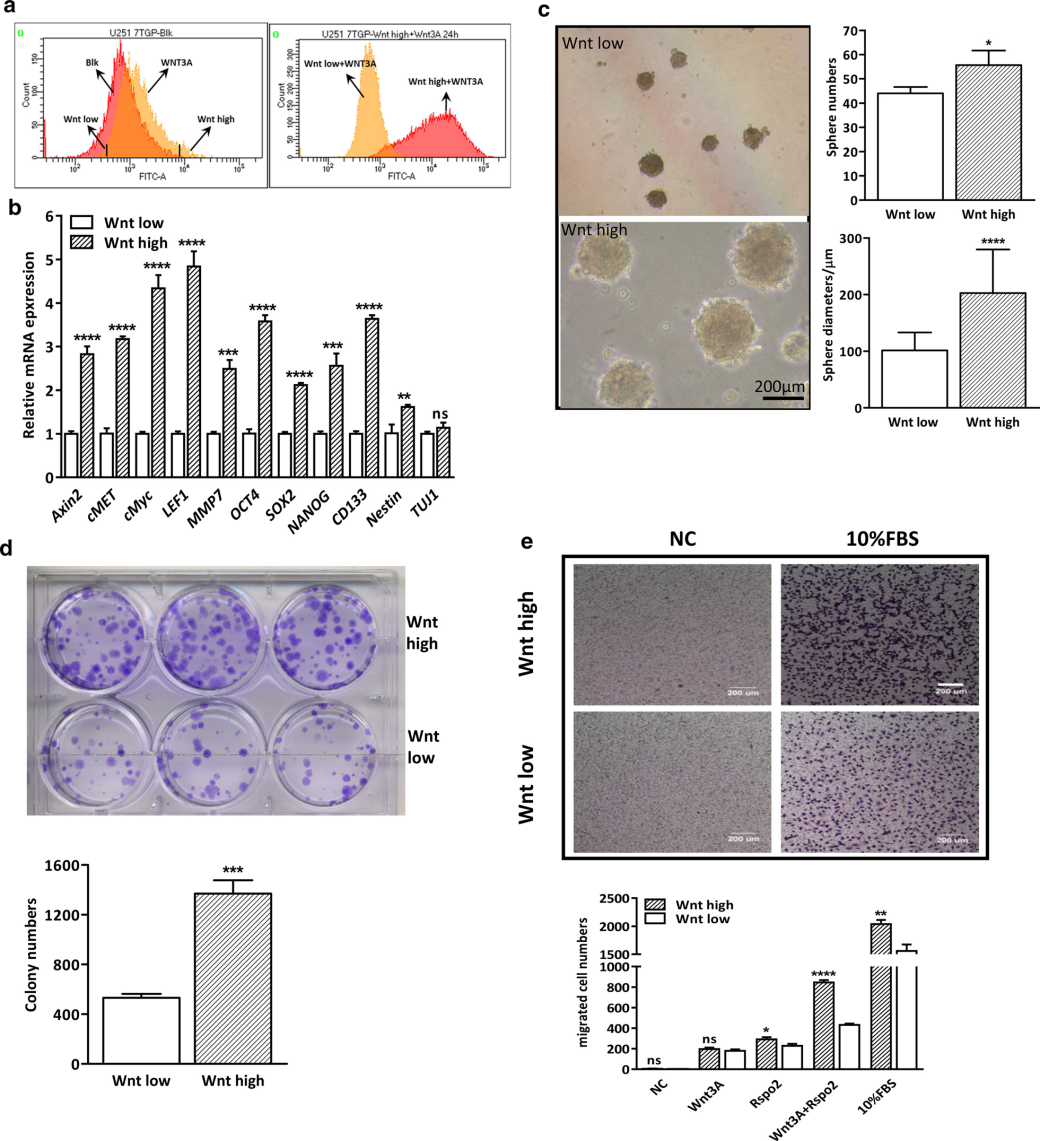
Wnt^high^ and Wnt^low^ sub-populations of U251 cells exhibit different cellular properties in vitro. **a** Isolation of Wnt^high^ and Wnt^low^ sub-populations of U251 cells. Left panel, Typical flow cytometry analysis depicting unsorted U251-7TGP cells treated with 20 ng/ml Wnt3A (yellow). For subsequent experiment, the 5% of cells with the highest and lowest GFP levels were sorted. Right panel, shown is the flow cytometric reanalysis of the respective subgroups after several rounds of enrichment. **b** Real-time PCR demonstrates that Wnt^high^ cells show higher expression levels of β-catenin targets and stem cell markers. **c** Sphere formation assay shows that Wnt^high^ cells have an enhanced sphere formation ability. Scale bar is 200 μm. Quantification analysis of data is expressed as the Mean ± SD from three independent experiments. **d** Soft agar assay shows Wnt^high^ cells have a higher anchorage-independent cell growth ability. Quantification analysis of data is expressed as the Mean ± SD from three independent experiments. **e** Transwell assay shows Wnt^high^ cells exhibit enhanced cell migratory ability. Quantification analysis of data is expressed as the Mean ± SD from three independent experiments. N. C. indicates vehicle control of Wnt^high^ and Wnt^low^ cells

Given that activation of Wnt/β-catenin signaling is instrumental for GSC traits, we reasoned that the different responsiveness of GBM cells to Wnt ligands could contribute to GSC property. To prove this hypothesis, U251 7TGP stable cells were cultured in serum-free medium containing 20 ng/ml Wnt3A for 24 h. Subsequently, Wnt^high^ cells were sorted from the highest 5% eGFP^+^ cells and Wnt^low^ cells were sorted from the lowest 5% GFP^+^ cells. After several rounds of enrichment, two subgroups of cells with distinctive response to Wnt3A were established (Fig. 5a). We then evaluated the canonical Wnt activity in the two cell subsets and found that the basal expression levels of the β-catenin targets (*Axin2*, *LEF1*, *cMyc*, *c*-*MET* and *MMP7*) were significantly increased in GFP/Wnt^high^ compared to GFP/Wnt^low^ cells, indicating a higher level activity of β-catenin signaling in Wnt^high^ cells (Fig. 5b). Of interest, we also found that Wnt^high^ cells displayed significantly increased expression levels of stemness genes including *OCT4*, *SOX2*, *NANOG*, *CD133* and *Nestin*, suggesting distinctive stemness state between Wnt^high^ and Wnt^low^ cells. In addition, comparative Western blotting in the two subpopulations corroborated high Wnt responsiveness in Wnt^high^ cells with upregulated expression of GFP, active β-CATENIN and β-catenin targets, such as AXIN2, c-MYC, CCND1, c-MET (Additional file 1: Fig S5A). In addition, the protein expression levels of EMT markers, such as TWIST and VIMENTIN, were also dramatically increased in the Wnt^high^ cells in response to Rspo2 and/or Wnt3A, suggesting distinctive malignancy states of the two subpopulations.

Having established the divergent Wnt/GFP status in Wnt^high^ and Wnt^low^ U251 cells, we went further to examine the biological properties of those two subpopulations. To our surprise, we did not find any significant difference in anchorage-dependent cell growth between Wnt^high^ and Wnt^low^ cells, either in untreated status or treated with Wnt3A and/or Rspo 2 (Additional file 1: Fig S5B). We then switched to sphere forming assay and the result showed that more spheres were formed in Wnt^high^ than that in Wnt^low^ cells. In addition, Wnt^high^ cells formed larger spheres than Wnt^low^ cells (Fig. 5c). Moreover, there were more colony formation with Wnt^high^ population than Wnt^low^ population in soft agar assay (Fig. 5d). We also determined their difference in cell migration and found that Wnt^high^ cells showed increased migratory capability compared to Wnt^low^ cells (Fig. 5e), which is in corroboration with the increased expression levels of TWIST and VIMENTIN in Wnt^high^ cells (Additional file 1: Fig S5A).

### Wnt/GFP^high^ population possesses increased tumorigenic potential in vivo

**Fig. 6:**
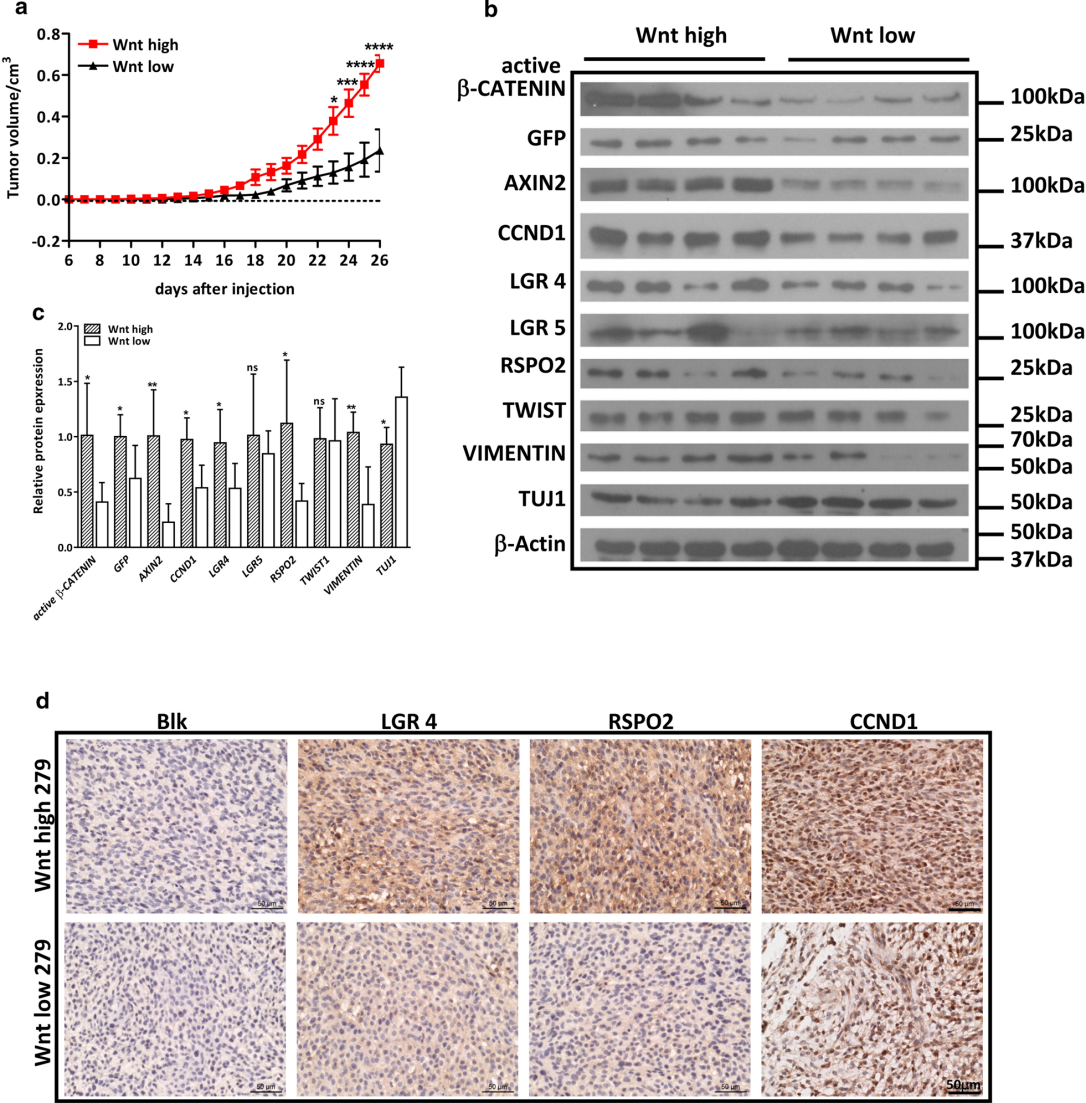
Wnt^high^ tumors grow faster and have high RSPO expression. **a** 1 × 10^4^ Wnt^high^ or Wnt^low^ cells were inoculated at the flanks of nude mice, and tumor growth curve were recorded. The result shows that Wnt^high^ cell-injected xenografts (n = 9) grow faster than Wnt^low^ cell-injected xenografts (n = 6). **b** Protein expression of β-catenin targets, RSPO/LGR, EMT markers and differentiation marker was compared between Wnt^high^ (n = 4) and Wnt^low^ (n = 4) xenografts. **c** Statistic analysis of G. **d** Tumor sections from Wnt^high^ or Wnt^low^ xenografts were stained by RSPO2, LGR4 and CCND1, where Wnt^high^ tumors display stronger staining compared to Wnt^low^ tumors. Scale bar is 200 μm

Next, we sought to determine whether difference in WNT responsiveness in GBM cells leads to altered tumorigenic capability in vivo. To do this, Wnt^high^ or Wnt^low^ subpopulations were subcutaneously implanted into 6 week old-male nude mice with two concentrations (Additional file 1: Fig S5C). The result showed that all mice formed tumors with 1 × 10^5^Wnt^high^ or Wnt^low^ cells. However, while all mice formed tumors with 1 × 10^4^ Wnt^high^ cells, only 6 out 9 mice formed tumors with 1 × 10^4^ Wnt^low^ cells (Additional file 1: Fig S5C). In addition, the tumors grew more slowly with 1 × 10^4^Wnt^low^ cells than that with Wnt^high^ cells (Fig. 6a). These results indicate that Wnt^high^ cells have increased tumorigenicity than Wnt^low^ cells in vivo. Subsequent closer examination of tumor samples demonstrated that the expression levels of activate β-CATENIN, eGFP and β-catenin target AXIN2, CCND1 were significantly higher in Wnt^high^ xenografts compared to Wnt^low^ xenografts, indicating high Wnt activity is maintained in Wnt^high^ cell-transplanted tumors. Correspondingly, we found a significantly higher expression of RSPO2 and LGR4 in these tumors as well (Fig. 6b–d). In addition, the expression levels of TWIST and VIMENTIN, which were indicative of higher migratory ability and GSC property, were significantly higher in Wnt^high^ xenografts. In contrast, the expression level of differentiation marker TUJ1 was significantly lower in Wnt^high^ xenografts compared to Wnt^low^ xenografts (Fig. 6b, c). Together, these data suggest a more malignant signature of Wnt^high^ xenografts.

## Discussion

Taken together, our study has unveiled a previously undefined role of Rspos in potentiating canonical Wnt signaling in GBM, which promotes cancer stemness trait.

In the current study, we have found that Rspo2 maintains GSC traits via potentiating Wnt. Wnt3A/Rspo2 promotes the self-renewal capacity of GSCs and prevents them from RA-or growth factor deprivation-induced differentiation (Fig. 4b–d, Additional file 1: Fig S4). These results clearly indicate that the stem-like characteristics of GSCs can be effectively maintained by the synergistic effect of Rspo2 and Wnt3A, while Rspo2 or Wnt3A alone is not sufficient. Our finding is consistent with the previous study showing that the Wnt–Lgr5–Rspo axis is crucial for maintaining colonic and pancreatic CSCs for long-term in vitro expansion and self-renewal [32, 34]. On the other hand, one recent study using lineage tracing clearly revealed that Wnt ligands had qualitatively distinct, non-interchangeable roles in intestinal stem cells (ISC) [42]. i.e. Wnt proteins themselves were unable to induce ISC self-renewal, but instead conferred a basal competency by enabling Rspo ligands to actively drive and specify the extent of stem-cell expansion. This functionally non-equivalent yet cooperative interaction mode between Wnt and Rspo may explain the synergistic effect of Wnt3A and Rspo2 on GSCs. Indeed, fine-tuning of Wnt/β-catenin activity by Wnt ligands is essential to optimally maintain neoplastic cells and the level of Wnt activation required for this effect is likely to be tissue and cell type specific [43]. It should be noted that GSCs are endowed with intrinsic high activity of Wnt-Lgr-Rspo signaling. In particular, the upregulation of Rspo/Lgr axis is prominent, i.e. Rspo2 and Lgr5 are upregulated more than 200- and 500fold respectively in U251 GSCs compared to parental cells (Fig. 3a–c). It is plausible that Rspo2 amplifies Wnt/β-catenin signaling and ensures a high and stable Wnt environment surrounding GSCs. Indeed, previous study has revealed that Rspo/Lgr signaling in ISCs is essential for establishing a Wnt gradient in the ISC niche [44].

Heterogeneous responsiveness to Wnt/β-catenin signaling has been observed in various cancers, such as colon cancer and breast cancer [45, 46]. Reciprocally, tumor cells with high β-catenin activity within a tumor mass appear to undergo EMT and acquire cancer stem cell property [47]. On the other hand, CSC properties can be altered by environmental cues, such as hypoxia and growth factor exposure. Interestingly, in the current study, we have shown that GBM cells exhibit distinctive Wnt ligand responsiveness. Wnt^high^ responders are more susceptible to extrinsic Wnt stimulation with both Wnt protein and Wnt enhancer. Treatment with Rspo2 alone or in combination with Wnt3A has mild effects on β-catenin targets in low-responder cells, whereas high-responders react boldly to Wnt3A, Rspo2, and to their combination (Additional file 1: Fig S6A). In addition, we have clearly demonstrated that Wnt^high^ responders exhibit a more aggressive malignant phenotype with stem cell traits than Wnt^low^ responders. First, the Wnt^high^ population expresses higher stemness genes and EMT markers compared to Wnt^low^ population. Second, Wnt^high^ cells show growth advantage in sphere formation assay and soft agar assay, indicating their enhanced stemness. Third, Wnt^high^ cells exhibit stronger migratory ability, suggesting a more aggressive phenotype. Finally, Wnt^high^ cells show enhanced tumorigenicity and grow faster than Wnt^low^ cells in vivo. In addition, Wnt^high^ xenografts are more malignant than Wnt^low^ xenografts. Altogether, these results clearly indicate that not only the baseline level of Wnt/β-catenin, but also the functional response and adaptability to contextual Wnt signals is of great importance for GBM stemness. In fact, the interaction between tumor cells and the surrounding microenvironment can locally affect the intracellular levels of canonical Wnt signaling, which triggers stemness, cell proliferation, EMT and invasive behavior. For instance, Rspo1, which is hormonally regulated in luminal epithelial cells by estrogen and progesterone, acts in concert with Wnt4 to expand mammary stem cells [48]. In addition, Rspo2 was reported to enhance Wnt signaling and stemness in Wnt responsive pancreatic cancer cells [34]. These findings indicate that both intrinsic and extrinsic factors are likely to play critical roles in cancer stemness, local invasion and metastasis by differentially modulating Wnt/β-catenin signaling. In our study, RSPO2 protein is found to be overexpressed in Wnt^high^ GBM xenografts (Fig. 6a–c), which confirms that Rspo2 plays a major role in regulating Wnt signaling in susceptible GBM cells. Nevertheless, it should be noted that RSPO2 and LGR4 are not globally expressed in all tumor cells in the Wnt^high^ GBM xenografts (Fig. 6d), suggesting heterogeneous property of GBM cells and their distinctive of β-catenin activity. Future investigation focusing on the link between the heterogeneous expression of RSPO/LGR and cancer stemness in primary GBM tissues is necessary.

During development, *Rspo2* is prominently expressed in the apical ectodermal ridge (AER) of the limb bud, and more expression has been detected in the developing lungs, brain, pharynx, teeth, long bones, craniofacial bones, and vertebrae [49]. The major phenotypes associated with the global loss of *Rspo 2* during development are limb and craniofacial malformations and hypomorphic lungs, which result in perinatal death [50, 51]. Rspo3 is highly expressed in the primitive streak during very early development and is also expressed in the developing neural tube, brain, limb bud, heart, kidney, and small intestine. However, embryonic death around E10 limits the investigation of further effects of *Rspo3* deficiency [52]. In our study, we observed that the mRNA expression levels of *RSPO2* and its receptors are relatively high in brain samples compared to that in GBM cell lines (Fig. 1a). The protein Atlas data in human cerebral cortex illustrates that RSPO2 is mostly expressed at the neurophil area that forms a synaptically dense region containing mostly unmyelinated axons, dendrites and glial cell processes with a relatively low number of cell bodies in the brain (https://www.proteinatlas.org). Noticeably, RSPO2 is almost undetectable in glial cells, which are the cell of origin of glioma. Thus, compared to the normal glial cells, RSPO2 is highly expressed in GBM cell lines. Given the well-established role of Wnt/β-catenin signaling in neural stem cell function and brain development, the regulatory effects of Rspos in neural stem cells warrant further investigation.

## Conclusions

Altogether, the present study has demonstrated that Rspo2 potentiates Wnt/β-catenin signaling in GBM. In addition, we have shown that not only the baseline level of Wnt signaling, but also the functional response and adaptability to contextual Wnt ligands is the determinant for GBM stemness and should be pursued as GSC readout. The findings of this study reveal an undefined role of Rspos in the development of GBM, and shed new light into the underlying molecular mechanisms contributing to GSC regulation, which may provide grounds for the development of therapeutic targets for GBM.

## Additional files

**Additional file 1: Figure S1.** Potentiation effect of Rspo2 in U87 cells. A-B, U87 cells were pre-treated in serum-free medium for 24 hours, then cultured in serum-free medium containing different WNT ligands for another 24 hours. Rspo2 shows potentiation effect of Wnt3A induced β-catenin targets (A) as well as RSPO2-LGRs (B). Blk indicates U87 cells cultured in 0.1% DMSO. (C) Schematic illustration of 7TGP vector. **Figure S2.** The effect of Wnt3A and/or Rspos on stem cell markers in U87 and U251 cells. A, mRNA expression levels of stem cell markers in U87 cells in respond to Wnt3Aand/or Rspo2. Blk indicates U87 cells cultured in 0.1% DMSO. B, Wnt 3A and Rspo2 do not affect CD133 expression in U251 cells. U251 cells were pre-treated in serum-free medium for 24 hours, then cultured in serum-free medium containing different Wnt ligands for another 24 hours. The cells were stained with CD133-APC antibody and analyzed for CD133 positivity by flow cytometry. Blk indicates U251 cells cultured in 0.1% DMSO. **Figure S3.** Establishment and Characterization of U251 and U87 GSCs. A, Flow cytometry analysis of U87 and U251 GSC-like cells. B, 5000 U251 cells or GSC-like cells were seeded in GSC medium for 10 days, sphere formation was evaluated for numbers and diameters. Quantification analysis of data is expressed as the Mean ± SD from three independent experiments. C, 200 U251 cells or GSC-like cells were used for holoclone assay, where U251 GSCs show an enhanced holoclone formation ability than normal glioma cells. D–E, U87 GSCs show upregulated mRNA expression levels of β-catenin targets (D), as well as RSPO-LGR genes (E). **Figure S4.** Rspo2/Wnt3A prevents RA and growth factor deprivation-induced differentiation in GSCs. A, all-trans retinoic acid (10 µM RA) was used to induce differentiation in U87 GSCs for 24 hours with or without WNT ligands (20 ng/ml). Real-time PCR was used to determine the effect on differentiation. Results show that Rspo2/Wnt3A treatment rescues RA-induced U87 GSC differentiation. Blk indicates GSCs cultured in DMEM with 0.1% DMSO. B, U251 GSCs were cultured in GSC media, or GSC media without EGF and FGF, or GSC media without EGF and FGF but with Wnt3A and Rspo2 for 7 days. Phase image shows the morphology of spheres. C, real-time PCR shows that Rspo2/Wnt3A treatment abolishes the downregulation of β-catenin targets caused by growth factor deprivation. Blk indicates U251 GSCs cultured in GSC media with 0.1% DMSO. **Figure S5.** Wnt^high^ and Wnt^low^ cell populations show different cellular behavior. A, Western blot analysis comparing the responsiveness of Wnt^high^ and Wnt^low^ cell populations. B, 3000 cells/well of U251 Wnt ^high^ and Wnt^low^ cells were pre-treated in serum-free medium for 24 hours, then cultured in serum-free medium containing different WNT ligands for another 4 days, and MTT assay was performed every 24 hours. C, Table shows serial dilution tumor inoculation assay using U251 Wnt^high^ and Wnt^low^ cells.

**Additional file 2: Table S1.** Primer used for realtime PCR.

**Additional file 3: Table S2.** Antibodies used in Western blot.
