# Supplementary material for: R-spodin2 enhances canonical Wnt signaling to maintain the stemness of glioblastoma cells
Source: Cancer Cell Int. 2018 Oct 11;18:156. doi: 10.1186/s12935-018-0655-3 (PMC6180579; doi:10.1186/s12935-018-0655-3)
Supplement: Supplementary file 1 — Additional file 1: Figure S1. Potentiation effect of Rspo2 in U87 cells. A-B, U87 cells were pre-treated in serum-free medium for 24 hours, then cultured in serum-free medium containing different WNT ligands for another 24 hours. Rspo2 shows potentiation effect of Wnt3A induced β-catenin targets (A) as well as RSPO2-LGRs (B). Blk indicates U87 cells cultured in 0.1% DMSO. (C) Schematic illustration of 7TGP vector. Figure S2. The effect of Wnt3A and/or Rspos on stem cell markers in U87 and U251 cells. A, mRNA expression levels of stem cell markers in U87 cells in respond to Wnt3Aand/or Rspo2. Blk indicates U87 cells cultured in 0.1% DMSO. B, Wnt 3A and Rspo2 do not affect CD133 expression in U251 cells. U251 cells were pre-treated in serum-free medium for 24 hours, then cultured in serum-free medium containing different Wnt ligands for another 24 hours. The cells were stained with CD133-APC antibody and analyzed for CD133 positivity by flow cytometry. Blk indicates U251 cells cultured in 0.1% DMSO. Figure S3. Establishment and Characterization of U251 and U87 GSCs. A, Flow cytometry analysis of U87 and U251 GSC-like cells. B, 5000 U251 cells or GSC-like cells were seeded in GSC medium for 10 days, sphere formation was evaluated for numbers and diameters. Quantification analysis of data is expressed as the Mean ± SD from three independent experiments. C, 200 U251 cells or GSC-like cells were used for holoclone assay, where U251 GSCs show an enhanced holoclone formation ability than normal glioma cells. D–E, U87 GSCs show upregulated mRNA expression levels of β-catenin targets (D), as well as RSPO-LGR genes (E). Figure S4. Rspo2/Wnt3A prevents RA and growth factor deprivation-induced differentiation in GSCs. A, all-trans retinoic acid (10 µM RA) was used to induce differentiation in U87 GSCs for 24 hours with or without WNT ligands (20 ng/ml). Real-time PCR was used to determine the effect on differentiation. Results show that Rspo2/Wnt3A treatment rescues RA-induced U [file 12935_2018_655_MOESM1_ESM.pptx]

## Slide 1
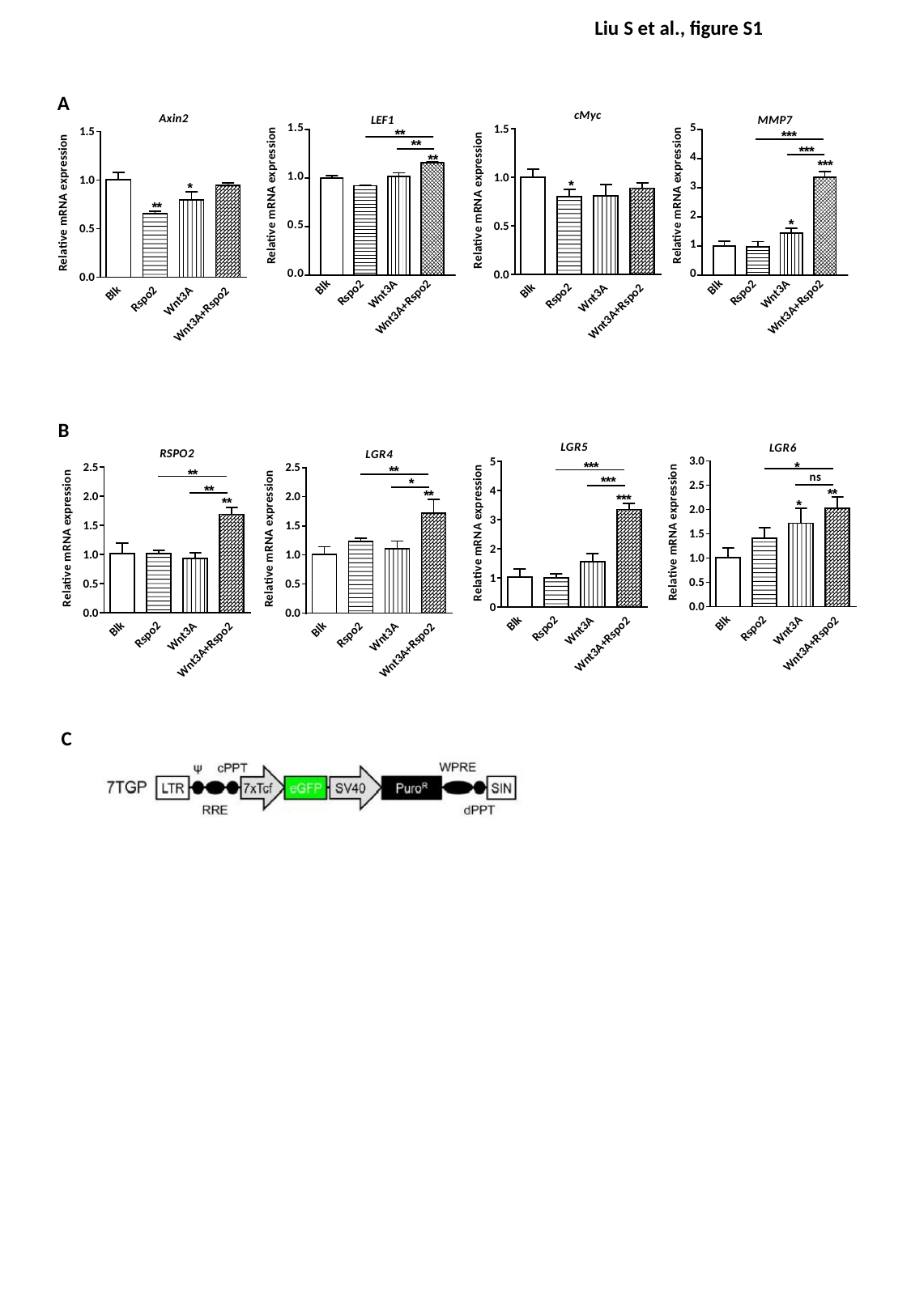

Liu S et al., figure S1
A
B
C

## Slide 2
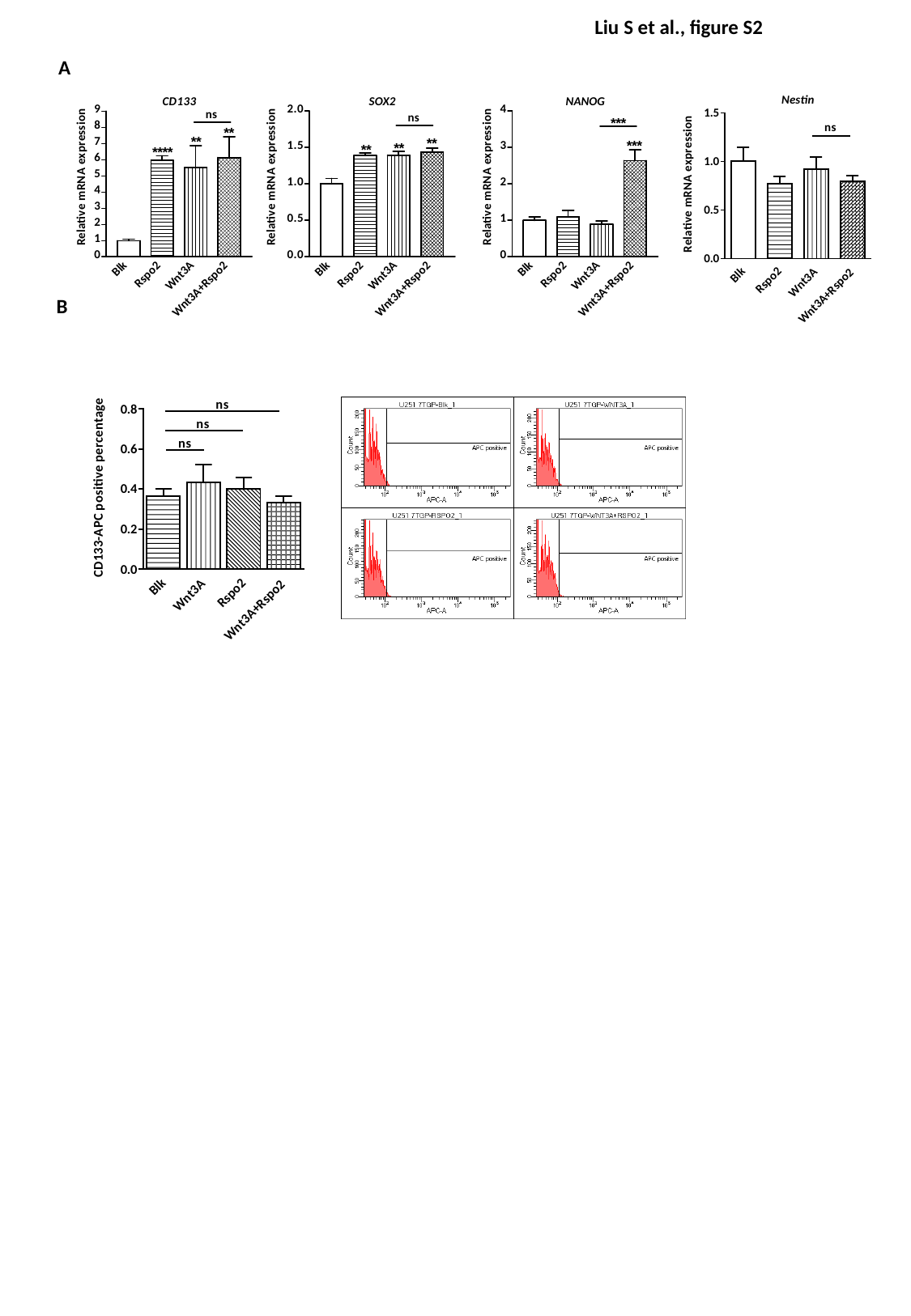

Liu S et al., figure S2
A
B

## Slide 3
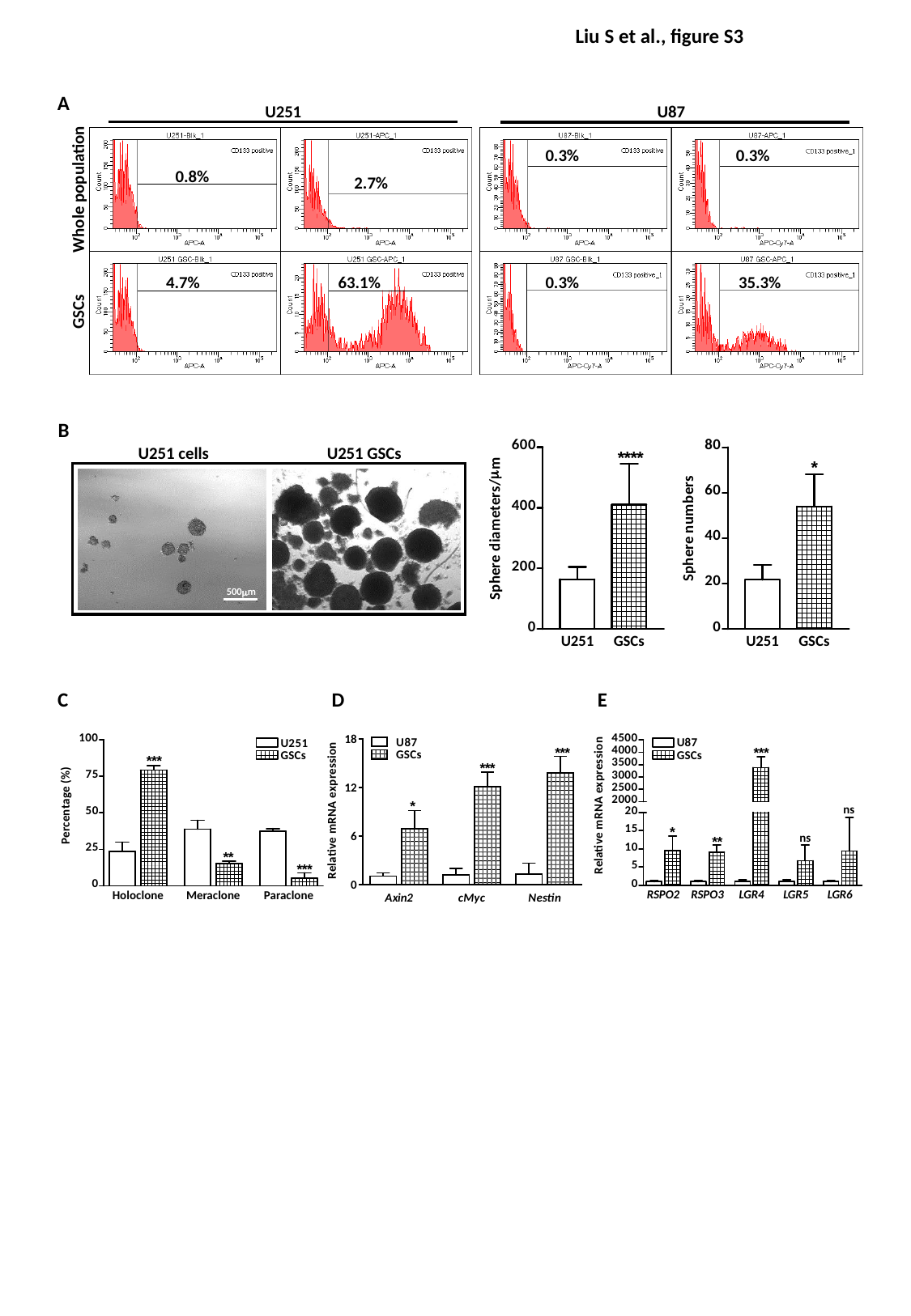

Liu S et al., figure S3
A
U251
U87
0.3%
0.3%
0.8%
2.7%
Whole population
35.3%
4.7%
63.1%
0.3%
GSCs
B
U251 cells
U251 GSCs
500μm
C
D
E

## Slide 4
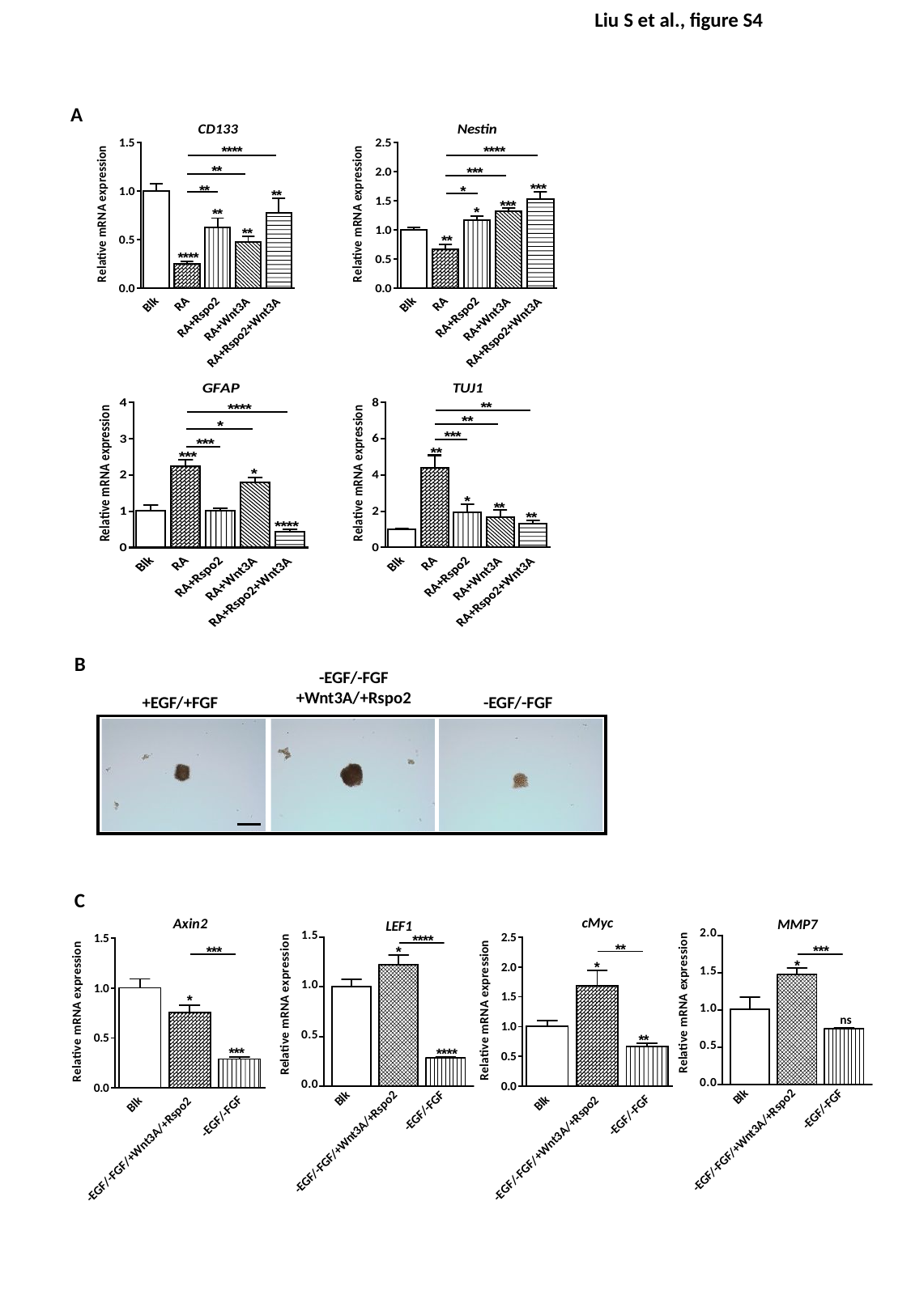

Liu S et al., figure S4
A
B
-EGF/-FGF
+Wnt3A/+Rspo2
+EGF/+FGF
-EGF/-FGF
C

## Slide 5
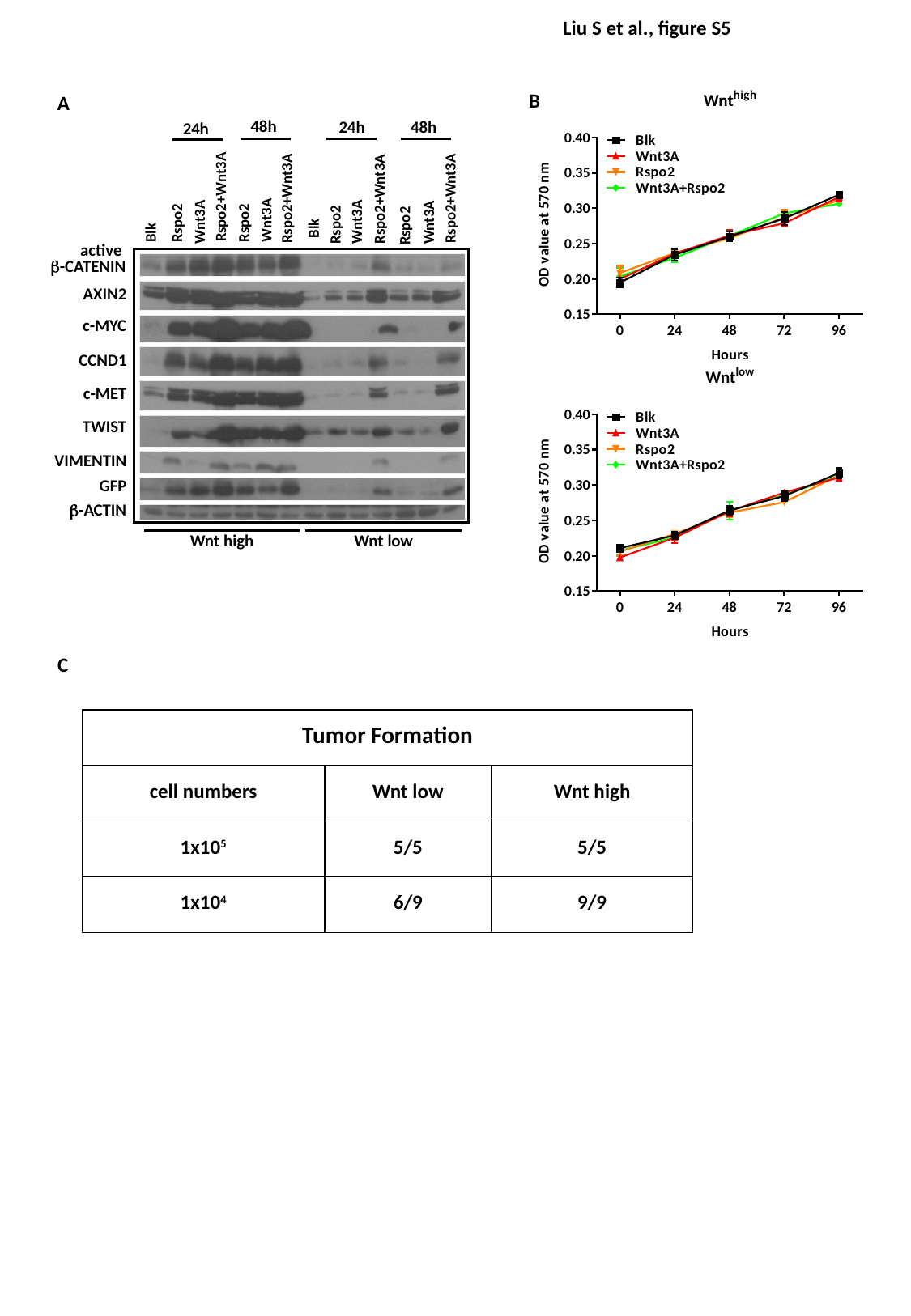

Liu S et al., figure S5
B
A
48h
24h
48h
24h
Rspo2+Wnt3A
Rspo2+Wnt3A
Rspo2+Wnt3A
Rspo2+Wnt3A
Wnt3A
Wnt3A
Wnt3A
Wnt3A
Rspo2
Rspo2
Rspo2
Rspo2
Blk
Blk
active
β-CATENIN
AXIN2
c-MYC
CCND1
c-MET
TWIST
VIMENTIN
GFP
β-ACTIN
Wnt high
Wnt low
C
| Tumor Formation | | |
| --- | --- | --- |
| cell numbers | Wnt low | Wnt high |
| 1x10^5^ | 5/5 | 5/5 |
| 1x10^4^ | 6/9 | 9/9 |
