# Supplementary material for: R-spodin2 enhances canonical Wnt signaling to maintain the stemness of glioblastoma cells
Source: Cancer Cell Int. 2018 Oct 11;18:156. doi: 10.1186/s12935-018-0655-3 (PMC6180579; doi:10.1186/s12935-018-0655-3)
Supplement: Supplementary file 2 — Additional file 2: Table S1. Primer used for realtime PCR. [file 12935_2018_655_MOESM2_ESM.docx]

Table S1

| Gene | Primer Sequence | Product Size |
| --- | --- | --- |
| OCT4 | F: 5’-GGGTTTTTGGGATTAAGTTCTTCA -3’  R: 5’-GCCCCCACCCTTTGTGTT -3’ | 62 |
| ACTB | F: 5’-GCGCTCAGGAGGAGCAAT -3’  R: 5’-GCACTCTTCCAGCCTTCCT -3’ | 227 |
| SOX2 | F: 5’-CAAAAATGGCCATGCAGGTT -3’  R: 5’-AGTTGGGATCGAACAAAAGCTATT -3’ | 62 |
| NANOG | F: 5’-ACAACTGGCCGAAGAATAGCA -3’  R: 5’-GGTTCCCAGTCGGGTTCAC -3’ | 110 |
| KLF4 | F: 5’-GAAATTCGCCCGCTCCGATGA -3’  R: 5’-CTGTGTGTTTGCGGTAGTGCC -3’ | 49 |
| PROM1/CD133 | F: 5’-CAGAGTACAACGCCAAACCA -3’  R: 5’-AAATCACGATGAGGGTCAGC -3’ | 245 |
| RSPO2 | F: 5’-TGGAAACCAGAACACGGCAA -3’  R: 5’-GTTCCTCTTCTCCTTCGCCTT -3’ | 146 |
| RSPO3 | F: 5’-TGTGCAACATGCTCAGATTACA -3’  R: 5’-TGCTTCATGCCAATTCTTTCCA -3’ | 83 |
| LGR4 | F: 5’-TCGAGGGCTGAGTGCTTTG -3’  R: 5’-ATGCCGTAACTGAACAAGTCC -3’ | 97 |
| LGR5 | F: 5’-CACCTCCTACCTAGACCTCAGT -3’  R: 5’-CGCAAGACGTAACTCCTCCAG -3’ | 94 |
| LGR6 | F: 5’-ACACAACCGCATCTGGGAAAT -3’  R: 5’-CGTTCCAGCTAAGATCCAGGG -3’ | 80 |
| MYC | F: 5’-TGCTGCCAAGAGGGTCAAGT -3’  R: 5’-GTGTGTTCGCCTCTTGACATTC -3’ | 118 |
| AXIN2 | F: 5’-ACTGCCCACACGATAAGGAG -3’  R: 5’-CTGGCTATGTCTTTGGACCA -3’ | 127 |
| LEF1 | F: 5’-CTTTATCCAGGCTGGTCTGC -3’  R: 5’-TCGTTTTCCACCATGTTTCA -3’ | 133 |
| MET | F: 5’-TGGTGCAGAGGAGCAATGG -3’  R: 5’-CATTCTGGATGGGTGTTTCCG -3’ | 110 |
| MMP7 | F: 5’-TGAATTTGGCCACTCTCTGGGTCT -3’  R: 5’-TCTGAATGCCTGCAATGTCGTCCT -3’ | 124 |
| NESTIN | F: 5’-GAAACAGCCATAGAGGGCAAA -3’  R: 5’-TGGTTTTCCAGAGTCTTCAGTGA -3’ | 167 |
| GFAP | F: 5’-CCTCTCCCTGGCTCGAATG -3’  R: 5’-GGAAGCGAACCTTCTCGATGTA -3’ | 161 |
| TUJ1 | F: 5’-ATGAGGGAGATCGTGCACAT -3’  R: 5’-GCCCCTGAGCGGACACTGT -3’ | 238 |
| SNAIL1 | F: 5’-GCTGCAGGACTCTAATCCAGA -3’  R: 5’-ATCTCCGGAGGTGGGATG -3’ | 83 |
| TWIST | F: 5’-GGACAAGCTGAGCAAGATTCAGA -3’  R: 5’-TCTGGAGGACCTGGTAGAGGAA -3’ | 73 |
| ZEB1 | F: 5’-GCCAACAGACCAGACAGTGTT -3’  R: 5’-TTTCTTGCCCTTCCTTTCTG -3’ | 94 |
