# Supplementary material for: R-spodin2 enhances canonical Wnt signaling to maintain the stemness of glioblastoma cells
Source: Cancer Cell Int. 2018 Oct 11;18:156. doi: 10.1186/s12935-018-0655-3 (PMC6180579; doi:10.1186/s12935-018-0655-3)
Supplement: Supplementary file 3 — Additional file 3: Table S2. Antibodies used in Western blot. [file 12935_2018_655_MOESM3_ESM.docx]

Table S2

| Antibody Name | Company* | Cat. No. | | Experiment# | Dilution |
| --- | --- | --- | --- | --- | --- |
| β tubulin | SC | sc-910 | | WB | 1:1000 |
| Mouse IgG1-APC | Miltenyi Biotec | 130-098-846 | | FACS | 1:10 |
| CD133/1 (AC133) | Miltenyi Biotec | 130-098-829 | | FACS | 1:10 |
| (Active) β-Catenin  (Ser33/37/Thr41) | CST | CST 8814S | | IF/ WB | 1:50 for IF  1:1000 for WB |
| Axin2 | Cell Signaling | 2151S | | WB | 1:1000 |
| cMyc | Cell Signaling | 5605 | | WB | 1:1000 |
| cyclin D1 | CST | sc-8396 | | WB | 1:1000 |
| GFP (N-terminal) | Sigma | G1544 | | WB | 1:1000 |
| LGR4 | SC | sc-390630 | | IF/ WB | 1:100 for IF  1:1000 for WB |
| GPR49 | Abcam | ab75732 | | IF/ WB | 1:100 for IF  1:1000 for WB |
| Mmp7 | CST | CST 3801 | | WB | 1:1000 |
| Mmp9 | Millipore | AB19016 | | WB | 1:1000 |
| Met | Cell Signaling | 8198S | | WB | 1:1000 |
| goat anti- mouse IgG- HRP | SC | sc-2005 | | WB | 1:1000 |
| goat anti- rabbit IgG- HRP | SC | sc-2357 | | WB | 1:1000 |
| Company* | | | CST: Cell Signaling Technology | | |
|  | | | SC: Santa Cruz Biotechnology | | |
| Experiment# | | | WB: Western blot | | |
|  | | | IF: Immunofluorescent staining | | |
|  | | | FACS: Flow Cytometry analysis | | |
